# Interesting Cases of Apoplexy

**Published:** 1830-04-01

**Authors:** 


### INTERESTING CASES OF APOPLEXY. By Dr. ABERCROMBIE.*

Much has been published on the pathology of apoplexy, and yet what obscurity still shrouds the rationale of many cases of that dreadful disease. Many well-informed professional men appear to imagine that organic lesions are always found after death to account for the symptoms and event. They talk, and too frequently act, as if extravasations or morbid accumulations of blood or serum, were the necessary and constant conditions to be met with. If an instance to the contrary is mentioned, it is met by scepticism, or the observation, that "had the dissector looked more sharply, something would have been found." This is a dangerous error, not only to the parties themselves but to their brethren, and one which we have done our best on several occasions to dispel. The following short and authentic case, detailed by Dr. Abercrombie, is calculated to make a strong impression on the minds of the gentlemen to whom we have alluded. It occurred under the care of Dr. Duncan, in the clinical ward of the Royal Infirmary of Edinburgh, in May, 1829.

*Case.* "A man, æt. 54, short-necked and of plethoric habit, was admitted into the Clinical Ward on 30th May. He was in a state of nearly perfect coma, speechless, and with palsy of the right side to such an extent, that even the intercostal muscles of that side did not act. The leg and arm of the left side were occasionally affected with convulsive motions. Breathing stertorous — deglutition much impaired. Pulse 74. The affection was of three days' standing, and had come on with vertigo—loss of vision—violent headach and vomiting.

"All the usual remedies were employed in the most judicious and active manner without benefit. On the 1st of June, there seemed to be a slight

---

* On the Brain. 2d Edit.

return of intelligence, but he soon relapsed into coma, and died on the 3d, without any change in the other symptoms.

"*Inspection.* A most minute and careful examination was made of the brain, without discovering any appearance of disease, except that the choroid plexus seemed rather darker than usual, and the basilar artery was diseased at one spot. By the side of the artery, there was a spot of the cerebral substance, no larger than a barleycorn, which appeared somewhat softened, but even this Dr. Duncan considered as extremely doubtful."

If we might trust our own observation, we would say that hemiplegia is more frequently connected with local pressure or structural alterations than general paralysis and coma. So many exceptions, however occur to these general rules, if rules they can be called, that they must always be received with reservation. This very day we witnessed the examination of a patient with disease of the heart, who had suffered from hemiplegia, and in whom there was nothing found to account for that affection. In another case which occurred during last Autumn, there was apoplexy, with some hemiplegia of the right side, and yet no satisfactory morbid appearances were discovered in the brain or elsewhere. The case was that of a gentleman who came over from Ireland to transact commercial business in town, was seized in the manner we have mentioned, and died in the course of four days. The dissection of the brain was conducted by Dr. Hodgkin, and it may well be believed that it was not done carelessly.

### II. RAPIDLY FATAL CEREBRAL DISEASE.

It is known by most practitioners, that instances of very sudden death are commonly dependent on diseases of the heart and great vessels rather than on apoplexy. But it must be recollected, that even the latter will occasionally prove fatal in almost as short a space of time as the rupture of an aneurismal sac of the aorta, or the snap of the vital chain in certain affections of the heart.

*Case.* "A woman, aged 54, who had been for several years liable to headach, attended a crowded meeting on the evening of 25th of June, 1829, and seemed in perfect health. Towards the conclusion of the meeting she uttered a loud and convulsive scream, and instantly fell down in a state of insensibility. She was immediately carried out and was seen by Dr. Macauley, who happened to be present; he found her pale and totally insensible, and the pulse feeble: and within five minutes from the first seizure she was dead.

"*Inspection.* The integuments of the head were much loaded with blood, On removing the dura mater, there was a thin but very extensive appearance of extravasated blood, or rather ecchymosis, which covered nearly the whole surface of the brain. In the substance of the anterior lobe of the right hemisphere there was a coagulum of blood the size of a large bean. All the other viscera were examined in the most accurate manner, but nothing was discovered, except a tubercle on the liver, and a small spot of ossification on the abdominal aorta."

### III. EXTRAVASATION IN THE TUBER ANNULARE AND IN THE THECA VERTEBRALIS.

When the extravasation takes place about the cerebellum, the symptoms appear to be more rapid than when it attacks the substance of the cerebrum. We think we have observed that pressure, if not considerable, about the pons varolii or medulla oblongata, is more apt to produce convulsions than when seated elsewhere. If, however, the pressure be great, convulsions do not occur, but the fatal issue is precipitated.

*Case.* "A gentleman, aged 37, had been for several months in bad health, being affected with occasional tightness of the chest and difficulty of breathing. He had also severe dyspeptic complaints, with occasional vomiting, a yellow tinge of his skin, and considerable uneasiness in the region of the liver. For these complaints he had been advised by his medical attendants in the north, to go to Cheltenham, and arrived in Edinburgh with that intention on 22d March, 1828. I saw him on the following day along with Mr. Wishart. We found his pulse frequent, his countenance sallow, and his expression febrile and anxious. He complained chiefly of tightness across his chest, with some pain in the region of the liver. Respiration was very imperfect along the right side of the thorax, and there was some œdema of the legs. By topical bleeding, purging, &c. he was considerably relieved; and on the 24th he expressed himself as feeling much better, but his pulse continued frequent. On the morning of the 25th he was suddenly seized with giddiness, noise and confusion in his head, and numbness of the whole right side. He was oppressed, but not comatose; answered questions distinctly, but in a loud voice, and with a peculiar manner. He complained chiefly of noise in his head, of a tight and cramped feeling of his right arm and leg, with much pricking and loss of command of the parts, but when desired to grasp another person's hand with his, the muscular power did not seem to be diminished. The expression of his countenance was vacant and fatuous: the eye was natural. The face was slightly distorted, and the speech was in some degree embarrassed. The pulse was 120."

After large bloodletting and the other usual remedies, the symptoms gradually assumed a more favourable aspect, and after four or five days, he was considered as being out of any immediate danger, though the effects of the attack were by no means removed. His pulse was now natural, his speech was distinct, and his mind entire; his sight was good, and the appearance of the eye natural, except a slight degree of paralysis of the upper eyelid of the right side. His breathing was easy, and he made no complaint, except of the tight and cramped feeling with numbness of the right arm and leg. His look, however, continued vacant and peculiar. His appetite and digestion were good, and his bowels easily regulated. He was improving in strength, and was able to be out of bed part of the day. This favourable state continued till the 14th of April, on which day he was found with a very frequent pulse without any other change in the symptoms. This febrile state continued on the two following days with rapid failure of strength, and he died on the evening of the 16th. He continued sensible to the last, and during this febrile attack, he seemed to have acquired an increased command over the limbs of the affected side. About the commencement of his illness of 25th March he complained of considerable uneasiness in passing his urine; for a day or two it was bloody, and there was a good deal of tenderness in the region of the bladder. After a few days this subsided, and he began to pass considerable quantities of puriform fluid of remarkable fetor, which subsided to the bottom of the chamberpot, after the urine had stood for a short time. This continued during the remainder of his life, though it had greatly diminished in quantity for several days preceding the last febrile attack. The urine was in sufficient quantity, and passed without difficulty.

"*Inspection.*—The brain and cerebellum were found in every respect in the most healthy state, and no vestige of disease was discovered until the cerebellum was separated from the tuber annulare. In doing so a cavity was exposed about the size of a large hazel nut, lined by a soft cyst, and full of dark grumous blood of a firm consistence. This remarkable cavity was formed partly in the substance of the tuber, and partly betwixt it and the base of the cerebellum. It was decidedly more to the left side than the right, and the surrounding substance was softened, and tinged with dark red points, as if from injection of dark blood. There was effusion in the thorax to the amount of at least lb. ii. The right lung was contracted and extensively hepatized; the left was much loaded with sero-purulent fluid. The liver was very considerably enlarged and of a pale-ash colour and granular texture. The left kidney was pale, indurated, and tubercular. The inner surface of the bladder was deeply injected, and in several places shewed distinct round ulcers about a quarter of an inch in diameter."

The foregoing is a very interesting case, but we regret that no mention is made of the state of the heart. The connexion, first pointed out by the French, between hypertrophy of the left ventricle and a morbid state of the vessels of the head, so frequent a concomitant or cause of apoplexy, is one that we have very frequently observed. Indeed we remember but two or three cases of opacity and induration of the basilars or carotids to any extent, unaccompanied by cardiac hypertrophy. On the other hand, when patients die of idiopathic hypertrophia of the heart it is extremely rare to find the vessels of the head or the aorta quite free from the atheromatous deposites. The subject is too extensive for us to enter into here, but we would earnestly intreat practitioners when investigating the morbid anatomy of apoplexy, to extend their researches to the heart and blood-vessels, instead of confining them merely to the brain. In the succeeding case the extravasation was very extensive.

*Case.* "A boy, aged 9, previously in perfect health, awoke in the night of 18th May, 1829, complaining of headach; had vomiting and slight convulsion. On the 19th, he was seen by Mr. W. Brown, who found him still complaining of headach with occasional vomiting, but without any urgent symptom. Under the usual treatment the complaint seemed gradually to subside, and on the 25th he appeared to be entirely recovered. But on the afternoon of that day, he had a return of convulsion, and in the evening complained much of headach. Pulse 64.—26th and 27th, said he was better, but seemed drowsy. Pulse slow. Bowels obstinate.—28th, had two attacks of convulsion, the second of which was very severe and continued for several hours; affecting chiefly the left side of the body. Pulse 130. On the 29th he was again better; but from this time he became gradually more and more drowsy, and at last comatose with squinting, and occasional convulsive motions of the limbs, and he died on the 3d of June. His death was preceded by severe convulsion of several hours duration. I saw him along with Mr. Brown from the 29th.

*Inspection.* — The surface of the brain was healthy. The lateral ventricles were distended with dark bloody fluid, and each of them contained a mass of coagulated blood; that in the right was the size of a large walnut, the other smaller. The 3d and 4th ventricles were quite filled with coagulated blood in a very firm state, and from the bottom of the fourth ventricle, the coagulum was traced outwards and spread along the base of the brain and cerebellum, and around the medulla oblongata. The spinal canal being now laid open, the dura mater of the cord appeared remarkably distended, and the cord was found through its whole extent entirely enveloped by a very firm and uniform stratum of coagulated blood. The brain and cord were in their substance healthy, and the source of the hæmorrhage could not be discovered."

Dr. Abercrombie remarks on the foregoing case that it presented the most extensive extravasation of blood which he has ever met with. It is also curious "from the period of life at which the affection took place, and its similarity in the symptoms to one of the common inflammatory affections terminating by effusion."

IV. Extensive Disease of the Left Hemisphere of the Brain.

The following is a good example of the kind of symptoms attendant on chronic disorganization of the brain. Scarcely ever amounting to evidence beyond conjectural, the practitioner is frequently thrown off his guard, and pronounces the case to be dyspepsia, hypochondriasis, or hysteria, till a paralytic or an apoplectic seizure rights the diagnosis in a melancholy way. The case to which we proceed was communicated to Dr. Abercrombie, by his able friend Dr. Kellie of Leith.

*Case.* "A medical gentleman, aged

56, of a cultivated mind and temperate habits, had been for some time liable to various ailments, which his medical friends considered as in a great measure hypochondriacal. The most defined complaints were occasional uneasiness in the site of the frontal sinus, and a very peculiar feeling of numbness in the point of the thumb. But his general health appeared good, and he was able to enter into all the usual enjoyments of life, having retired from practice, till he was one day seized, while walking, with sudden sickness and faintness. These were followed by some headach, and an obvious difficulty of articulation, or rather a difficulty in finding the expression which he wished to make use of. He was now treated by bleeding and the other usual means; but this peculiar loss of the recollection of words continued and gradually increased, so that he had greater and greater difficulty in recollecting the words which he meant to employ, but he had no difficulty in pronouncing them. His understanding at this time was quite entire; his pulse varying from 80 to 112. He was nearly confined to the house, but out of bed during the day; and all the usual remedies were employed in the most assiduous manner. After he had gone on in this way for several weeks, he began to have slight distortion of the mouth, and complained of numbness of the right arm, and soon after of weakness of the right leg. These symptoms gradually increased to perfect hemiplegia; and about this time, also, he entirely lost his speech. He was now confined to bed, but without coma. He had the perfect use of his sight and hearing, and, as far as could be judged, his understanding was entire. He died with symptoms of bronchitis in the ninth week from the first attack.

*Inspection.*—The left hemisphere of the brain was found to be diseased throughout in a very singular manner. Some parts of the mass were indurated, others softened; and it presented a variety of colours, chiefly a rose-colour, grey, and yellow; and the more diseased portions were highly vascular. In some places there were distinct insulated masses, inclosed in vascular cysts; these were generally indurated, but some were softened, and they were of a rose or flesh colour passing into grey. The change from those parts which retained a natural appearance to these degenerated portions was abrupt, and marked by a rose-coloured line. These rose-coloured portions were chiefly in the parts nearest the surface; in the central parts this passed into the yellow or the grey, and many portions were in a state of ramollissement. The whole left hemisphere, in fact, presented little else than a mass of concentric indurations and softenings of the various colours which have been mentioned. On the upper part of the hemisphere, the disease did not extend entirely to the surface of the convolutions; but at the base of the anterior and middle lobes it extended to the surface, and at one place there was a well defined spot of superficial ulceration the size of a split pea."

A few more articles will enable us to get through the additional cases in this edition of Dr. Abercrombie's work. The use we are making of it proves the estimation in which we hold it, and were the plan of our author adopted generally in the construction of medical writings, the public, the profession, and the science of medicine would be greatly the gainers. Nothing remunerates us for the unpleasant task of scraping the tinsel from the productions of professed book-makers, save the pleasure we experience in meeting with works of genuine merit. Would Dr. Abercrombie wish us to say more?

